# Climate Change Modulates Multitrophic Interactions Between Maize, A Root Herbivore, and Its Enemies

**DOI:** 10.1007/s10886-021-01303-9

**Published:** 2021-08-20

**Authors:** Anouk Guyer, Cong van Doan, Corina Maurer, Ricardo A. R. Machado, Pierre Mateo, Katja Steinauer, Lucie Kesner, Günter Hoch, Ansgar Kahmen, Matthias Erb, Christelle A. M. Robert

**Affiliations:** 1 Institute of Plant Sciences, University of Bern, Altenbergrain 21, 3013 Bern, Switzerland; 2 Oeschger Centre for Climate Change Research (OCCR), University of Bern, Falkenplatz 16, 3012 Bern, Switzerland; 3 Department of Environmental Sciences - Botany, University of Basel, Schönbeinstrasse 6, 4056 Basel, Switzerland

**Keywords:** Climate change, Multitrophic interactions, Maize, Root herbivory, Entomopathogenic nematodes

## Abstract

How climate change will modify belowground tritrophic interactions is poorly understood, despite their importance for agricultural productivity. Here, we manipulated the three major abiotic factors associated with climate change (atmospheric CO_2_, temperature, and soil moisture) and investigated their individual and joint effects on the interaction between maize, the banded cucumber beetle (*Diabrotica balteata*), and the entomopathogenic nematode (EPN) *Heterorhabditis bacteriophora*. Changes in individual abiotic parameters had a strong influence on plant biomass, leaf wilting, sugar concentrations, protein levels, and benzoxazinoid contents. Yet, when combined to simulate a predicted climate scenario (Representative Concentration Pathway 8.5, RCP 8.5), their effects mostly counter-balanced each other. Only the sharp negative impact of drought on leaf wilting was not fully compensated. In both current and predicted scenarios, root damage resulted in increased leaf wilting, reduced root biomass, and reconfigured the plant sugar metabolism. Single climatic variables modulated the herbivore performance and survival in an additive manner, although slight interactions were also observed. Increased temperature and CO_2_ levels both enhanced the performance of the insect, but elevated temperature also decreased its survival. Elevated temperatures and CO_2_ further directly impeded the EPN infectivity potential, while lower moisture levels improved it through plant- and/or herbivore-mediated changes. In the RCP 8.5 scenario, temperature and CO_2_ showed interactive effects on EPN infectivity, which was overall decreased by 40%. We conclude that root pest problems may worsen with climate change due to increased herbivore performance and reduced top-down control by biological control agents.

**Supplementary Information:**

The online version contains supplementary material available at 10.1007/s10886-021-01303-9.

## Introduction

Climate change represents a considerable pressure on living organisms to swiftly adapt to new environmental conditions. Shifts in plant and animal physiology, behavior, and phenology, can further reverberate onto their interaction networks. In particular, climate change is expected to strongly reconfigure multitrophic interactions between plants, insect herbivores, and their enemies (Harvey and Malcicka 2015; Rosenblatt and Schmitz 2016; Chidawanyika et al. 2019; Damien and Tougeron 2019; Han et al. 2019).

Climatic factors, such as CO_2_, temperature, and precipitation, influence plant molecular functions, developmental processes, physiology, and morphology (Gray and Brady 2016). For instance, elevated temperature and CO_2_ can stimulate photosynthetic carbon rate assimilation, decrease nitrogen contents, and increase biomass production (Ainsworth and Long 2005; Sage and Kubien 2007; Robinson et al. 2012), although the observed effects are stronger in C3 than in C4 plants (Ghannoum et al. 2000). On the other hand, drought stress can counterbalance and even revert these effects (Reich et al. 2014). Furthermore, elevated CO_2_, increased temperature, and drought stress generally stimulates the production of plant secondary metabolites (Bidart-Bouzat and Imeh-Nathaniel 2008; Nguyen et al. 2016a, b; Ehlers et al. 2020). Overall, changes in climatic conditions explain up to 39% of yield variability in crops (Ray et al. 2015).

CO_2_, temperature, and precipitation, also influence insect herbivore development, mobility, fecundity, and survival (Gregory et al. 2009; Khaliq et al. 2014). For instance, warming accelerates insect development and generation numbers (Ayres and Lombardero 2000). Climate-mediated changes in host plant nutritional quality and defenses generally result in increased consumption rates (Hamann et al. 2021). Thus, climate change will likely favor pest outbreaks through direct and plant-mediated effects (Deutsch et al. 2018).

Current models also predict that climate change will impair the performance, behavior, and survival of herbivore enemies (Voigt et al. 2003; Thakur 2020). Elevated CO_2_ can disrupt the ability of natural enemies to process cues from their environment (Draper and Weissburg 2019). Increasing temperatures enhance parasitism success until an optimum temperature, temperatures exceeding this optimum leads to a decline in parasitism efficiency (Furlong and Zalucki 2017; Chidawanyika et al. 2019). As the optimum temperature is lower for individuals of the third trophic level than for their insect host/prey, the formers are likely more susceptible to global warming (Furlong and Zalucki 2017). Precipitation patterns directly modulate the physiology and foraging behavior of herbivore enemies (Jamieson et al. 2012; Barnett and Facey 2016; Torode et al. 2016; van Doan et al. 2021). Climatic factors can also affect the third trophic level through changes in lower trophic level quality. For instance, elevated atmospheric CO_2_ levels induce plants to produce higher concentrations of plant secondary metabolites (Bezemer and Jones 1998; Bidart-Bouzat and Imeh-Nathaniel 2008), in turn decreasing the herbivore quality as a host or a prey (Harvey et al. 2005; Lampert et al. 2011). Several studies predict that climate change may therefore benefit herbivore outbreaks through a loss of top-down control by natural enemies (Stireman et al. 2005; Harvey et al. 2020).

Interestingly, while soil shelters major crop pest insects (Hunter 2001), the impact of climate change on belowground food webs remains poorly understood (Staley and Johnson 2008; McKenzie et al. 2013; Hiltpold et al. 2016). Bale et al. suggested that the direct effects of increasing temperatures may be buffered in soil due to the dense environment (Bale et al. 2002). However, plant-mediated effects of temperatures may arise. For example, elevated temperatures increase plant evapotranspiration and thus decrease soil moisture in the rhizosphere (Norby and Luo 2004; Dermody et al. 2007). Reduced soil moisture can considerably alter the survival and abundance of soil organisms directly, although the direction and strength of the effects depends on the organism taxonomic classification and feeding guild (Chaves et al. 2003; Pacchioli and Hower 2004; Johnson et al. 2010; Rohde et al. 2010; Torode et al. 2016; Guyer et al. 2018; van Doan et al. 2021). CO_2_ levels are higher in soil than in the atmosphere, mostly due to root respiration and microbial processes (Haimi et al. 2005). It is therefore assumed that the direct effects of elevated atmospheric CO_2_ levels on the soil fauna will be minor, although it could disrupt the foraging strategies of some herbivore insects or enemies (Zhang et al. 2021; Guerenstein and Hildebrand 2008). Atmospheric CO_2_ levels indirectly affect root feeders and their enemies through plant-mediated effects. For instance, elevated CO_2_ reduces plant stomatal conductance, resulting in an increased soil moisture in the vicinity of the roots (Cowan and Farquhar 1977). Recently, Hiltpold et al. (2020) further highlighted that elevated CO_2_ reconfigure maize root architecture and reduced the recovery rate of entomopathogenic nematodes from the rhizosphere. Documenting the impact of climate change on belowground trophic interactions will complement current predictive models and allow the development of efficient, sustainable, pest management strategies to ensure food production in the coming decades,

Yet, a current limitation to predict how climate change will impact food-webs is the limited number of studies that manipulate multiple (more than two) abiotic factors associated with climate change simultaneously, while respecting natural conditions, including day/night cycles (Scherber et al. 2013; Rosenblatt et al. 2016). To date, little is known about the interactive impact of multiple parameters on trophic cascades (van der Putten et al. 2010; Robinson et al. 2012; Kreyling and Beier 2013; Hiltpold et al. 2016; Jactel et al. 2019). The impact of combined changes in CO_2_, temperature, and precipitation patterns cannot be extrapolated from the impact of each factor alone. Climatic variables affect plants, herbivores, and their natural enemies through additive and interactive (synergistic or antagonistic) effects (Darling and Côté 2008; Anderson and Song 2020). For instance, the combination of heat and drought triggers a larger detrimental effect on plant growth and yield than each of the factors individually (Mittler 2006). The combination of elevated CO_2_, increased temperature, and drought leads to variable plant responses depending on the plant species, soil type, nutrient availability, management, and spatial distribution (Erda et al. 2005; Moss et al. 2010; Challinor et al. 2014; Leng and Huang 2017; He et al. 2018; IPCC 2018; Reich et al. 2018). A meta-analysis conducted on the impact of different climatic variables and their interactions onto herbivore and predator survival demonstrated the high frequency of non-additive effects, including synergistic and antagonistic interactions, of combined climatic variables (Darling and Côté 2008). Similarly, non-additive effects between two climatic factors were reported for predation or parasitism success. For example, while elevated temperature and drought individually enhance the success of a parasitoid wasp, their combination led to inverted effects (Romo and Tylianakis 2013). Additional multifactorial studies, simultaneously manipulating multiple climatic factors, are required to generate more reliable models about the impact of climate change on trophic cascades (Gregory et al. 2009).

Here, we take a step towards filling this gap of knowledge by manipulating atmospheric CO_2_, temperature, and soil moisture, as well as the presence of root herbivores, and their natural enemies in the plant environment. We measured markers of plant growth and metabolism, herbivore performance and survival, as well as enemies’ efficiency in controlling a herbivore population. We used an agricultural model involving maize plants, the root herbivore *Diabrotica balteata*, and entomopathogenic nematodes *Heterorhabditis bacteriophora*. Maize, a C4 plant, is one of the most important cultivated crop plants worldwide, and root feeders of the *Diabrotica* species are important pests and cause severe economic yield losses (Johnson et al. 2016; Marchioro and Krechemer 2018). Entomopathogenic nematodes are commonly used as biological control agents to control root herbivore populations in the field (Toepfer et al. 2005; Hiltpold et al. 2010). We hypothesized (i) that climate change would strongly reconfigure the plant primary and secondary metabolism through interactive effects of temperature and moisture, (ii) that herbivores would mostly be affected by climatic variables through indirect, plant-mediated, effects, and (iii) that entomopathogenic nematodes would suffer from direct and indirect synergistic impacts of elevated CO_2_, temperature, and drought. Understanding how climate change will shape bottom-up and top-down effects on herbivorous insects will help developing further sustainable pest management strategies.

## Methods and Materials

### Biological Resources

Maize seeds (*Zea mays*, cv. Quattro, Delley Seeds and Plants Ltd, Delley, Switzerland) were soaked in tap water for 12 h and planted into 1 L plastic pots filled with 1.2 kg (± 10 g) field soil (Landerde, Ricoter, Aarberg, Switzerland). Field soil (40% sand, 35% silt, 25% clay) was sieved through a 2 cm mesh before planting. Because implementing the climatic treatments on germinating seedlings was lethal for all seedlings exposed to drought, all plants were first grown in a greenhouse (temperature 20 °C ± 2 °C, soil moisture 30%, light 16:8 h L/D) for 2 weeks prior to the start of the experiment.

Eggs of the cucumber beetle, *Diabrotica balteata* LeConte were kindly provided by Oliver Kindler (Syngenta, Stein, Switzerland). The larvae were reared on germinated maize seedlings (Hybrid 44110, Delley Seeds and Plants Ltd, Delley, Switzerland) until use. Wax moths, *Galleria mellonella* were bought at a fishing shop (Fischereibedarf, Bern, Switzerland). Entomopathogenic nematodes (EPNs), *Heterorhabditis bacteriophora*, strain EN01, were bought from Andermatt Biocontrol (Grossdietwil, Switzerland) and reared in *G. mellonella*. Emerging infective juveniles were stored for 7 days at 10 °C before use.

### Current and Predicted Climatic Conditions

Current conditions were determined according to climatic data from the Swiss Central Plateau (Average of conditions in June between 2005 and 2017, 15 cm depth, Oensingen, Switzerland) provided by MeteoSwiss (Federal Office of Meteorology and Climatology, Zürich, Switzerland). Obtained current conditions were 400 ppm atmospheric carbon dioxide (CO_2_), 17.4 °C soil temperature, and 27.0% soil gravimetric water content. Predicted conditions correspond to the Representative Concentration Pathway 8.5 (RCP, Intergovernmental Panel on Climate Change (IPCC) report for central Europe (Collins et al. 2013)). RCP 8.5 is an extreme-case scenario in which CO_2_ emissions continue to rise until 2100. Under this scenario, atmospheric CO_2_ concentrations would increase to 800 ppm CO_2_, the average soil temperature would be 20.8 °C (+ 3.4 °C), and soil moisture would decrease to 22.2% (− 17.8% precipitation) by 2100. An intermediate moisture level of 24.6% (− 8.7% precipitation) was further included in full factorial experiments. Linear correlations between daily air and soil temperatures, as well as between monthly precipitation and soil moisture allowed the calculations of predicted soil temperature and moisture (van Doan et al. 2021). Soil temperatures followed a diurnal variation of 3 °C. The minimal temperature was reached at 6 am and the maximal temperature achieved at 4 pm.

### Plants, Herbivores and EPN Exposure to Current and Predicted Abiotic Conditions

Two-week old plants were subjected to different combinations of current and predicted RCP 8.5 abiotic factors. Overall, we combined two levels of CO_2_ (Current: 400 ppm, RCP 8.5: 800 ppm), two levels of soil temperatures (Current: 17.4 °C, and RCP 8.5: 20.8 °C), and three levels of soil moisture (Current: 27%, Intermediate: 24.6%, RCP 8.5: 22.2%), in a full factorial design (12 combinations).

The plants were placed in four glass phytotrons (Phytotrons, type US75DU-Pi-5, Weiss Technik, Altendorf, Switzerland) set at different temperatures and CO_2_ levels. Briefly, the phytotrons CO_2_ levels were set to either 400 ppm or 800 ppm. The temperatures were established following a diurnal cycle ranging between 13.4 °C at night and 18.4 °C during the day or between 16.8 °C at night and 21.8 °C to reach soil temperatures corresponding to current and RCP 8.5 scenarios respectively. The soil temperatures were verified using a soil data logger (Temperature/Humidity UBS Datalogger RHT10, Extech instruments, MA 02451, USA) placed in one control pot per phytotron. Soil moisture conditions were obtained by daily watering the plants with different volumes of water. Water deficits were calculated based on pot weights and considering initial pot mass (soil dry weight and pot mass) and an estimation of the plant biomass according to their size (Guyer et al. 2018, Online Resource 1). Using this method, the water contents of low (RCP 8.5), intermediate, and current precipitation levels were 22.1 (± 0.22), 24.3 (± 0.25), and 26.6 (± 0.28)% respectively. The air moisture was not controlled. To avoid a phytotron bias, plants were rotated between phytotrons two times a week, and the conditions within the phytotrons were re-adjusted according to the plant treatment. One week after initiating exposure to the different abiotic conditions, half of the plants of each treatment were infested with six second-instar larvae of the herbivore *D. balteata*. The larvae were placed in two 5 cm depth holes 2 cm away from the maize stem. The two holes were immediately filled with soil again. One week later, 2500 EPNs were added to half of the control and half of the herbivore-infested plants within each combination of abiotic conditions. In total, the experiment, therefore, accounted for 48 treatments (n = 4–5 per treatment). The different abiotic conditions were maintained until plant harvest. The latter was conducted 37 days after planting. Soil moisture was adjusted to 30% water content in all treatments 2 h before collection. Due to the magnitude of the experiment, the plants were harvested on three consecutive days.

### Plant Response to Biotic and Abiotic Factors

Plant wilting was assessed twice per plant, respectively 1 and 2 days before harvest. The two scores, ranging from 1 (no symptom) to 4 (severe leaf rolling), were averaged for further analyses. Leaf and root fresh weights were measured upon harvest. Herbivore damage was evaluated on individual roots as follows: one insect bite was scored 10, one insect tunnel was scored 50 and a fully damaged/removed root was scored 100. The maximum score per root was 100. The individual root scores were averaged within each root system, resulting in one score per plant. The roots were then flash frozen in liquid nitrogen and stored at − 80 °C for further analyses. The root biochemical response to biotic and abiotic factors was assessed by grinding 100 mg of frozen root tissue and measuring soluble sugars, proteins, and benzoxazinoids. Soluble sugars (glucose, fructose, sucrose) were extracted and quantified as described by Machado et al. (2013). Soluble proteins were extracted in 20 mM Tris lysis buffer and analyzed with a Bradford method, using a Coomassie Plus (Bradford) Assay Kit (Thermo Scientific, USA). Benzoxazinoids were extracted in 1 mL acidified MeOH: H_2_O (50:50 v/v; 0.1% formic acid) and analyzed with an Acquity UHPLC-MS system as previously described (Hu et al. 2018b).

### Herbivore Performance

All herbivore larvae were collected from the root systems and soil. The number of recovered larvae was used as a proxy for herbivore survival. All collected larvae were weighed.

### EPN Infectivity

EPN infectivity was measured by baiting EPNs from the soil. Briefly, aliquots of homogenized soil (80 g/pot) were added into solo cups (1 oz Sovereign Shot Glasses, Maryland Plastic, Inc. China). Five *G. mellonella* larvae were placed in the cups for 7 days. As EPN-infected larvae turn red after 3–5 days post-infection (Fenton et al. 2011), the infection status of the wax moth larvae could be visually assessed.

### Statistical Analyses

Statistical analyses were performed with R (R version 3.4.3) using R studio (Rstudio version 1.1.442). The experiment followed a fully multifactorial design and response variables were analyzed using linear models, with soil moisture, temperature, CO_2_ levels, *D. balteata* infestation, and EPN-inoculation as fixed effects. Response variables of current and RCP 8.5 conditions were analyzed with a subset of data using linear models. All models were tested visually for normality and equality of variance using the package RVAideMemoire (Hervé 2018). Starting from all possible interactions, the model was stepwise reduced until single effects and significant interactions remained. ANOVA analysis was used to analyze the effects of response variables. Comparisons of means were performed using Tukey’s HSD test (p ≤ 0.05).

The influence of different abiotic parameters on plant, herbivore, and natural enemy performance was analyzed with three structural equation models, using the lavaan R package (Rosseel 2012). Analysis of direct and indirect effects of root herbivore performance (larval survival and root damage) and climate parameters on root biomass accumulation was performed with *D. balteata* infested pots (n = 120). The influence of direct and indirect effects of host plant chemistry (soluble sugars and benzoxazinoids) and climate parameters on root herbivore survival in presence versus absence of EPNs was analyzed using a multigroup approach (n_−EPN_ = 60, n_+EPN_ = 60). The influence of direct and indirect effects of maize physiology (soluble proteins, soluble sugars, and root biomass) and climate parameters on EPN infectivity accumulation was analyzed with EPN-treated pots (n = 120). The quality of the SEMs was assessed with χ^2^ goodness of fit test (p-value > 0.05 indicates that SEM fits the data), root mean square error of approximation value (RMSEA), and the comparative fit index (CFI). All statistical results and model fit indices can be found in Online Resources 2 and 3.

## Results

### Climate Change Effects on the Plant Metabolism

**Fig. 1:**
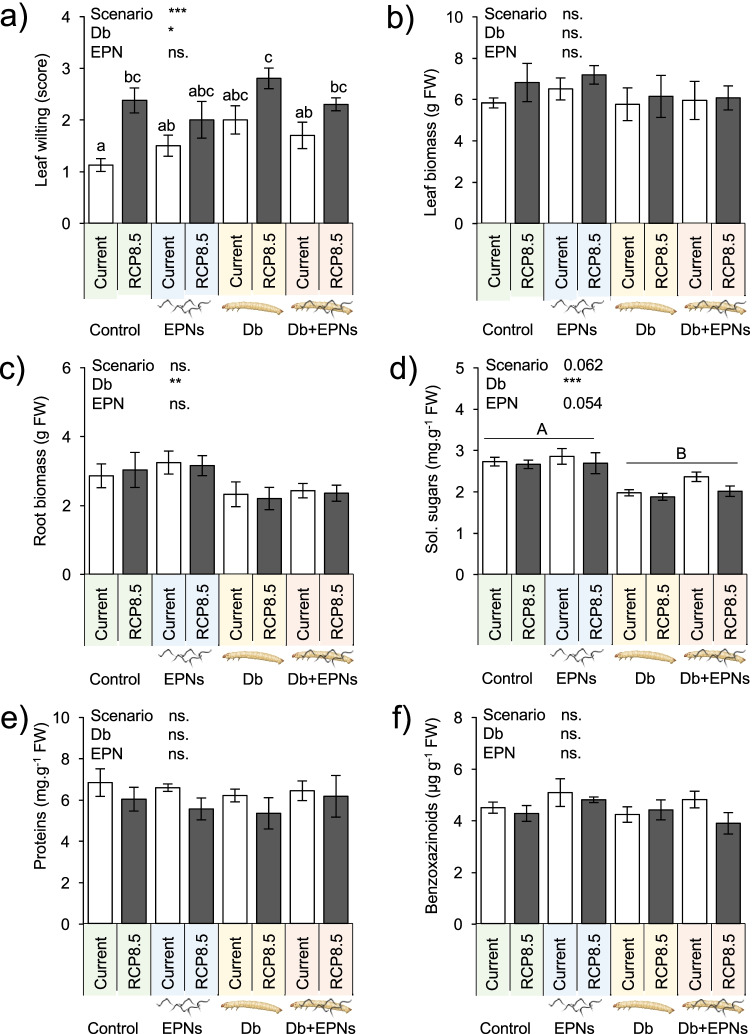
Maize responses to the root herbivore and its enemies under current and predicted climatic scenario RCP 8.5 (Collins et al. 2013). **a** Leaf wilting (scored from 1: no symptom to 4: severe leaf rolling), **b** leaf fresh biomass, **c** root fresh biomass, **d** root soluble sugar contents, **e** root soluble protein contents, **f** root benzoxazinoid concentrations. Current conditions: 400 ppm CO_2_, 17.4 °C, and 27.0% soil gravimetric water (“current”). RCP 8.5 conditions: 800 ppm CO_2_, 20.8 °C, and 22.2% soil gravimetric water. Plants subjected to herbivory were infested with six second-instar larvae of the herbivore *Diabrotica balteata*. A week later, half of the control and half of the herbivore-infested plants further received 2500 entomopathogenic nematodes, *Heterorhabditis bacteriophora*. Average ± SEM are shown. Stars indicate a significant impact of the tested treatment (linear model). *p ≤ 0.05, **p ≤ 0.01, ***p ≤ 0.001). No interaction between treatments was noted. Different letters indicate significant differences (Tukey’s HSD: p ≤ 0.05). All statistical results are shown in Table 1

In RCP 8.5 conditions, maize plants exhibited stronger leaf wilting symptoms than under current conditions (Fig. 1a). The predicted climatic scenario did not impair maize leaf and root fresh mass, root soluble sugar and protein contents, nor root secondary metabolite concentrations (Fig. 1b-f). Root herbivory by *D. balteata* increased leaf wilting and decreased root, but not leaf, biomass (Fig. 1a-c). Herbivory further led to differences in root soluble sugar concentrations, but not in soluble protein nor benzoxazinoid contents (Fig. 1d-f). The presence of entomopathogenic nematodes tended to increase soluble glucose concentrations in roots (Fig. 1d). Interestingly, no significant interactions between climatic scenarios, herbivory, or natural enemy presence were detected.

**Fig. 2:**
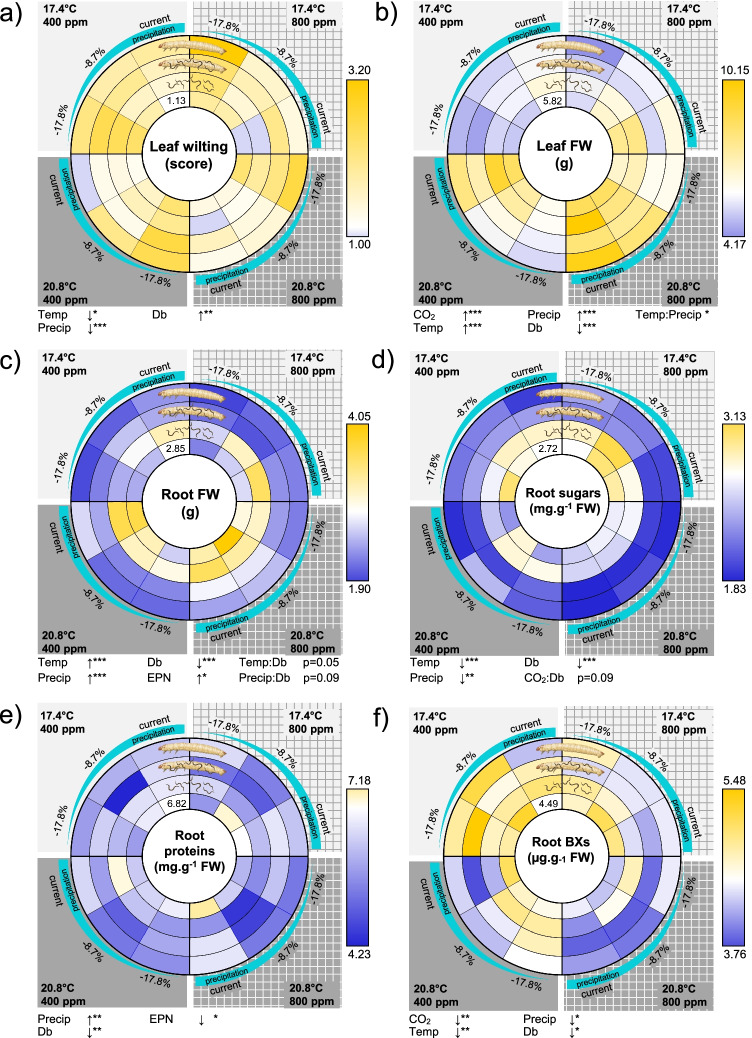
Maize responses to individual and combined abiotic and biotic factors. The plant responses to the full factorial 48 combinations of CO_2_ (current: 400 ppm, RCP 8.5: 800 ppm), temperature (current: 17.4 °C, RCP 8.5: 20.8 °C), moisture (current: 27% gravimetric moisture, intermediate − 8.7% precipitation relative to current conditions (24.6% gravimetric moisture), RCP 8.5: − 17.8% precipitation relative to current conditions (22.2% gravimetric moisture)), root herbivory by *Diabrotica balteata* (Db), and presence of the entomopathogenic nematodes (EPNs), *Heterorhabditis bacteriophora*, as natural enemies of the herbivore, are presented with a spider net. The color code indicates the relative deviation of the averages from current climate conditions (white indicates current control conditions, blue and yellow respectively indicate reductions and increases of the responsive variable). The vertical separation indicates ambient versus elevated atmospheric CO_2_ conditions. The horizontal separation indicates high versus low soil temperatures. Each quarter follows a soil moisture gradient, corresponding to different precipitation predictions (current, − 8.7% and − 17.8%). Different circle layers correspond to the different biotic conditions, starting with the control treatments in the center (plant only), followed by plants + EPNs, plants + Db + EPNs, and plants + Db. **a** Leaf wilting (scored from 1: no symptom to 4: severe leaf rolling), **b** leaf fresh biomass (FW), **c** root fresh biomass (FW), **d** root soluble sugar contents, **e** root soluble protein contents, **f** root benzoxazinoid concentrations (BXs). *Temp* temperature, *Moist* moisture, *Db* *Diabrotica balteata* (herbivore), *EPNs* entomopathogenic nematodes (herbivore enemies). The p-values of the treatment effects and significant interactions are indicated (*p ≤ 0.05, **p ≤ 0.01, ***p ≤ 0.001). The arrows indicate the direction of the response along the treatment gradient. All statistical results are shown in Table 1

**Fig. 3:**
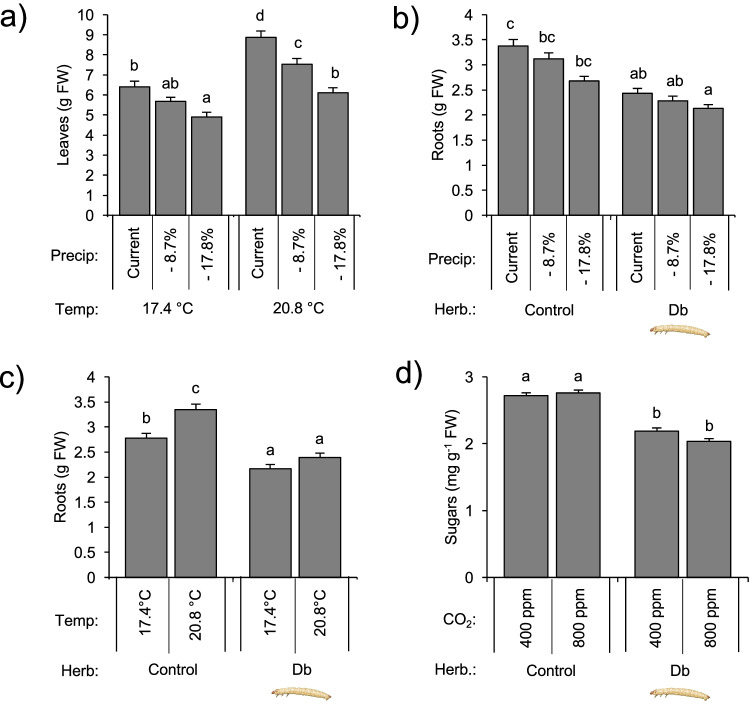
Plant responses to interactive effects (p < 0.10) between biotic and abiotic factors. **a** Leaf fresh weight is modulated by the interaction between soil temperature and moisture, **b** root fresh weight is modulated by the interaction between soil moisture and root herbivory *Diabrotica balteata*, **c** root fresh weight is modulated by the interaction between temperature and root herbivory *D. balteata*, and **d** soluble sugar contents are modulated by the interaction between CO_2_ and herbivory. Average ± SEM are shown. *Herb*. herbivory treatment, *Temp*. temperature, *Precip*. precipitation. Different letters indicate significant differences (Tukey’s HSD: p ≤ 0.05). All statistical results are shown in Table 1

CO_2_, temperature, and moisture showed additive effects in shaping plant development and metabolism (Fig. 2, Table 1). Elevated CO_2_ levels increased the leaf biomass and reduced benzoxazinoid (DIMBOA-Glc and DIM_2_BOA-Glc) contents in roots (Fig. 2, Online Resource 3). Elevated temperature resulted in lower leaf wilting, increased leaf and root fresh masses, and lower concentrations of fructose and glucose, but not of sucrose (Fig. 2, Online Resource 3). Decreased soil moisture levels mostly led to the opposite effects, including increased leaf wilting, reduced leaf and root fresh masses, and higher fructose, glucose, protein, and benzoxazinoid (DIM_2_BOA-Glc) concentrations (Fig. 2, Online Resource 3). CO_2_ did not show any interactive effects with temperature nor moisture. The combination of elevated temperature and decreased moisture resulted in additive effects on all factors, except for leaf biomass as the impact of drought was increased under elevated temperature (Fig. 3a).

**Table 1:** Summary from ANOVAs (1a) and F values (1b) of individual and interactive treatment effects on plant-, herbivore-, and nematode response traits

	CO_2_	T	M	Db	EPN	T:M	CO_2_:M	CO_2_:T	T:Db	M:Db	CO_2_:Db
*(a)*											
Wilting (%)		↓*	↓***	↑**							
FW leaves (g)	↑***	↑***	↑***	↓***		●*					
FW roots (g)		↑***	↑***	↓***	↑*				○ ^0.055^	○ ^0.093^	
Sugars (mg g^−1^ FW crown root)		↓***	↓**	↓***							○ ^0.087^
Protein (mg g^−1^ FW crown root)			↑**	↓***	↓*						
BXDs (µg g^−1^ FW crown root)	↓**	↓**	↓*	↓*							
Root damage (%)		↑^0.065^				○^0.084^					
*D. balteata* weight (mg per larva)	↑***	↑***									
*D. balteata* survival (%)		↓***			↓***	○^0.051^	○^0.096^				
Soil infectivity (%)	↓***	↓***	↓*					●*	○^0.083^		
*(b)*											
Wilting (%)	1.916	**4**.**663**	**105**.**801**	**11**.**093**	0.150						
FW leaves (g)	**14**.**720**	**77**.**925**	**68**.**737**	**16**.**339**	0	**6**.**109**					
FW roots (g)	0.876	**17**.**254**	**18**.**543**	**73**.**815**	**4**.**783**				3.7258	2.841	
Sugars (mg g^−1^ FW crown root)	1.039	**44**.**322**	**9**.**648**	**292**.**544**	1.782						**4**.**017**
Protein (mg g^−1^ FW crown root)	0.391	0.989	**9**.**520**	**8**.**235**	**4**.**260**						
BXDs (µg g^−1^ FW crown root)	**7**.**509**	**10**.**949**	**4**.**682**	**6**.**748**	0.607						
Root damage (%)	0.102	3.479	2.257		0.892	3.032					
*D. balteata* weight (mg per larva)	**20**.**161**	**13**.**128**	1.969		0.303						
*D. balteata* survival (%)	0.049	**13**.**133**	0.192		**39**.**520**	3.876	2.811				
Soil infectivity (%)	**16**.**358**	**89**.**057**	**4**.**860**	0.553				**4**.**273**	3.057		

Arrows indicate the direction of response along the treatment gradient, significant interactions are indicated with solid points and trends are indicated with open points

*T* temperature, *M* moisture, *CO*_*2*_ atmospheric CO_2_, *Db* *D. balteata*, *EPN* entomopathogenic nematodes, *FW* fresh weight, *BXDs* benzoxazinoids

p-values are indicated (*p ≤ 0.05, **p ≤ 0.01, ***p ≤ 0.001)

Bold values indicate significant effects (*p *<0.05)

Root herbivory impact under the RCP 8.5 scenario was mostly the result of additive effects with abiotic factors, except on root biomass and sugar contents, which resulted in slight, albeit non-significant, interactions with CO_2_, temperature, and moisture. Root herbivory increased leaf wilting and overall decreased leaf and root biomass (Fig. 2). The positive effects of elevated temperature, as well as the negative effects of reduced moisture on the root biomass, tended to fade away upon herbivory (Fig. 3b, c). Root herbivory reconfigured the plant metabolism by increasing root sucrose levels and by decreasing root glucose and fructose concentrations (Fig. 2, Online Resource 3). Elevated CO_2_ levels accentuated the decrease in glucose and fructose contents upon herbivory (Fig. 3d, Online Resource 3).

CO_2_, temperature, and moisture did not affect the plant response to entomopathogenic nematodes. Interestingly the presence of EPNs in the soil was sufficient to enhance the root biomass and decrease root protein contents (Fig. 2). These effects did not depend on abiotic factors.

### Climate Change Effects on Herbivore Performance

**Fig. 4:**
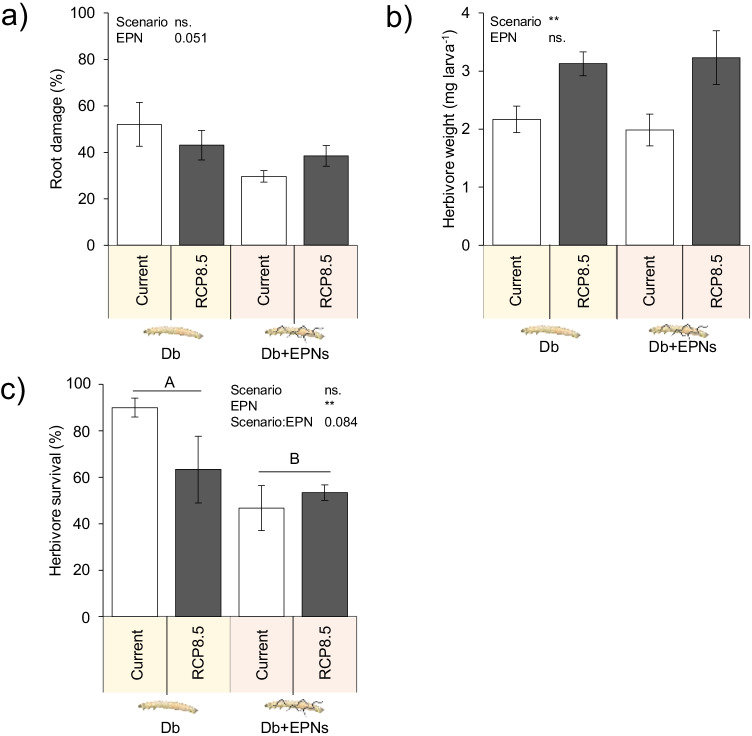
Herbivore performance under current and predicted climatic scenario RCP 8.5 (Collins et al. 2013). **a** Root damage caused by the soil-dwelling herbivore *Diabrotica balteata*. Herbivore damage was evaluated on individual roots as follow: one insect bite was scored 10, one insect tunnel was scored 50 and a fully damaged/removed root was scored 100. The average score per plant was calculated. **b** Average individual herbivore mass, and **c** herbivore survival. Average ± SEM are shown. Current conditions: 400 ppm CO_2_, 17.4 °C, and 27.0% soil gravimetric water (“current”). RCP 8.5 conditions: 800 ppm CO_2_, 20.8 °C, and 22.2% soil gravimetric water. Plants were infested with six second-instar larvae of the herbivore *Diabrotica balteata*. A week later, half of the control and half of the herbivore-infested plants further received 2500 entomopathogenic nematodes (+EPNs) or not (−EPNs), *Heterorhabditis bacteriophora*. Average ± SEM are shown. Stars indicate a significant impact of the tested treatment (linear model). *p ≤ 0.05, **p ≤ 0.01, ***p ≤ 0.001). No interaction between treatments was significant. Different letters indicate significant differences (Tukey’s HSD: p ≤ 0.05). All statistical results are shown in Table 1

RCP 8.5 conditions increased the herbivore weight gain but did not affect root damage (Fig. 4a, b). Predicted RCP 8.5 conditions did not alter the herbivore survival (Fig. 4c).

**Fig. 5:**
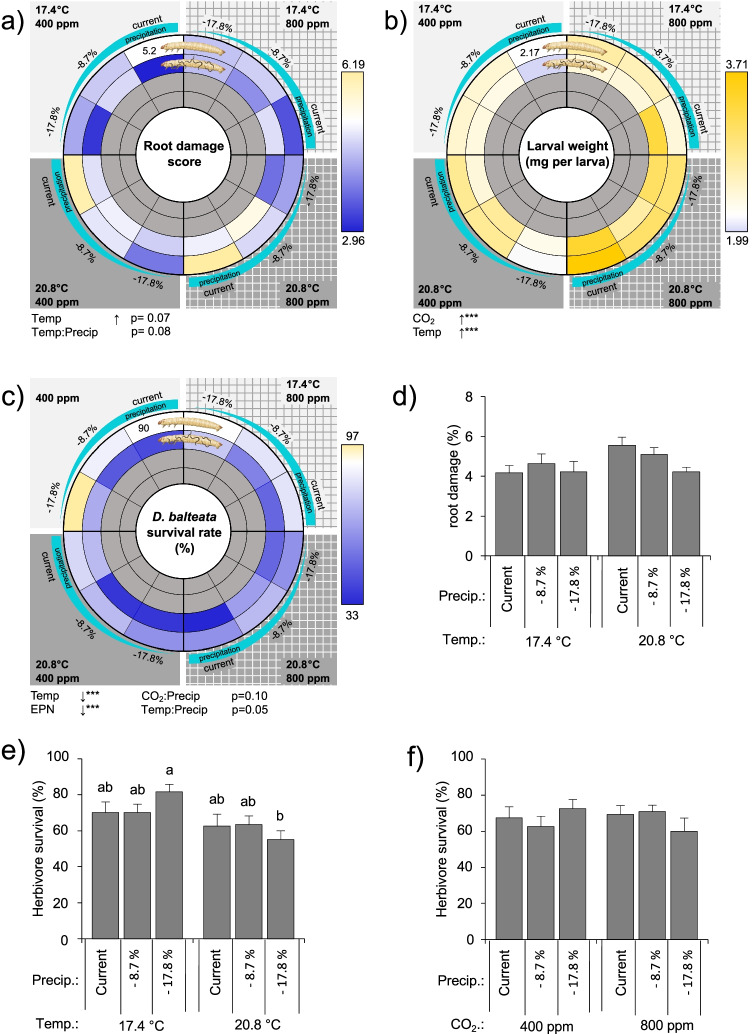
Herbivore performance in response to individual and combined abiotic and biotic factors, and to their interactions. The responses of the root herbivore, *Diabrotica balteata*, to the full factorial 24 combinations of CO_2_ (current: 400 ppm, RCP 8.5: 800 ppm), temperature (current: 17.4 °C, RCP 8.5: 20.8 °C), moisture (current: 27% gravimetric moisture, intermediate − 8.7% precipitation relative to current conditions (24.6% gravimetric moisture), RCP 8.5: − 17.8% precipitation relative to current conditions (22.2% gravimetric moisture)), and presence of the entomopathogenic nematodes (EPNs), *Heterorhabditis bacteriophora* are presented with a spider net. The color code indicates the relative deviation of the averages from current climate conditions (white indicates current control conditions, blue and yellow respectively indicate reductions and increases of the responsive variable). The vertical separation indicates ambient versus elevated atmospheric CO_2_ conditions. The horizontal separation indicates high versus low soil temperatures. Each quarter follows a soil moisture gradient, corresponding to different precipitation predictions (current, − 8.7% and − 17.8%). Different circle layers correspond to the different biotic conditions, starting with the control treatments in the center (plant only), followed by plants + EPNs, plants + Db + EPNs, and plants + Db. Plants from only the two outer circles were infested by herbivores, thus the inner circles are shown in grey. **a** Root damage caused by the herbivore. Herbivore damage was evaluated on individual roots as follow: one insect bite was scored 10, one insect tunnel was scored 50 and a fully damaged/removed root was scored 100. The average score per plant was calculated, **b** average individual herbivore mass, **c** herbivore survival, interactive effects of temperature and moisture on **d** root damage and **e** herbivore survival, and **f** of CO_2_ and moisture on herbivore survival. *Temp*. temperature, *Precip*. precipitation, *EPNs* entomopathogenic nematodes (herbivore enemies). The p-values of the treatment effects and significant interactions are indicated (*p ≤ 0.05, **p ≤ 0.01, ***p ≤ 0.001). The arrows indicate the direction of the response along the treatment gradient. All statistical results are shown in Table 1

CO_2_, temperature, their interactions with soil moisture, and the presence of EPNs determined the root herbivore performance and survival (Table 1). Elevated CO_2_ levels increased the herbivore weight gain (Fig. 5b). Increased temperatures increased root damage, enhanced herbivore mass, but decreased herbivore survival (Fig. 5a–c). Drought tended to decrease herbivore survival and damage, but only when drought was combined to elevated temperature or CO_2_ levels (Fig. 5d-f). Temperature and CO_2_ showed additive effects. Yet, both interacted with soil moisture, which alone did not affect the tested herbivore performance parameters.

**Fig. 6:**
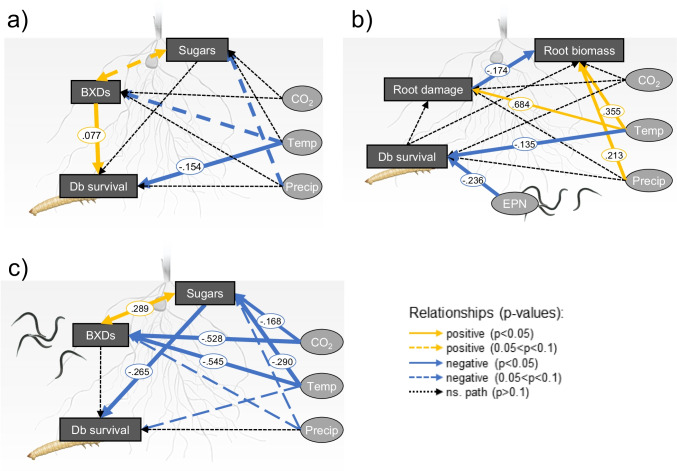
Root herbivore survival modulation by direct and indirect effects of climate change. Structural equation models of the relationships between major climate parameters (CO_2_, soil temperature and precipitation), plant responses and herbivore survival. **a** Survival of the root herbivore, *Diabrotica balteata*, as a function of climatic factors, root biomass, and presence of entomopathogenic nematodes *Heterorhabditis bateriophora* (EPNs). Survival of the root herbivore *D. balteata* as a function of climatic factors, root sugar and benzoxazinoid concentrations in **b** absence and **c** presence of EPNs in the rhizosphere. Single terms were included only. Arrow weights are proportional to standardised coefficients, which are indicated alongside the arrow. Blue arrows: negative relationships (continuous: p < 0.05, dashed: 0.05 < p < 0.10), yellow arrows: positive relationships (continuous: p < 0.05, dashed: 0.05 < p < 0.10), black dashed arrows: non-significant relationships, improving the models. *Temp* temperature, *Precip* precipitation, *Db* *Diabrotica balteata* (herbivore), *EPN* entomopathogenic nematodes. Model fit indices can be found in Online Resource 2

To evaluate whether the abiotic parameters affected the root herbivore directly or through changes in host plant chemistry, we constructed SEMs for larval performance and survival in the presence and absence of EPNs. SEM on performance significantly differed from the data and is therefore not shown. In absence of EPNs, increased temperatures directly reduced *D. balteata* survival (Fig. 6a). Increased temperature further tended to decrease root benzoxazinoid levels, which were unexpectedly positively correlated with herbivore survival (Fig. 6a). EPN infectivity was a major determinant of herbivore survival (Fig. 6b). In presence of EPNs in soil, no direct effects of the abiotic parameters were detected to affect the herbivore survival (Fig. 6c). Instead, increased temperature and CO_2_ suppressed root soluble sugars, which decreased herbivore survival (Fig. 6c, Online Resource 4). Thus, indirect effects on plant primary metabolism are determinants for herbivore survival in presence of natural enemies.

### Climate Change on Entomopathogenic Nematode Efficacy as Biological Control Agents

**Fig. 7:**
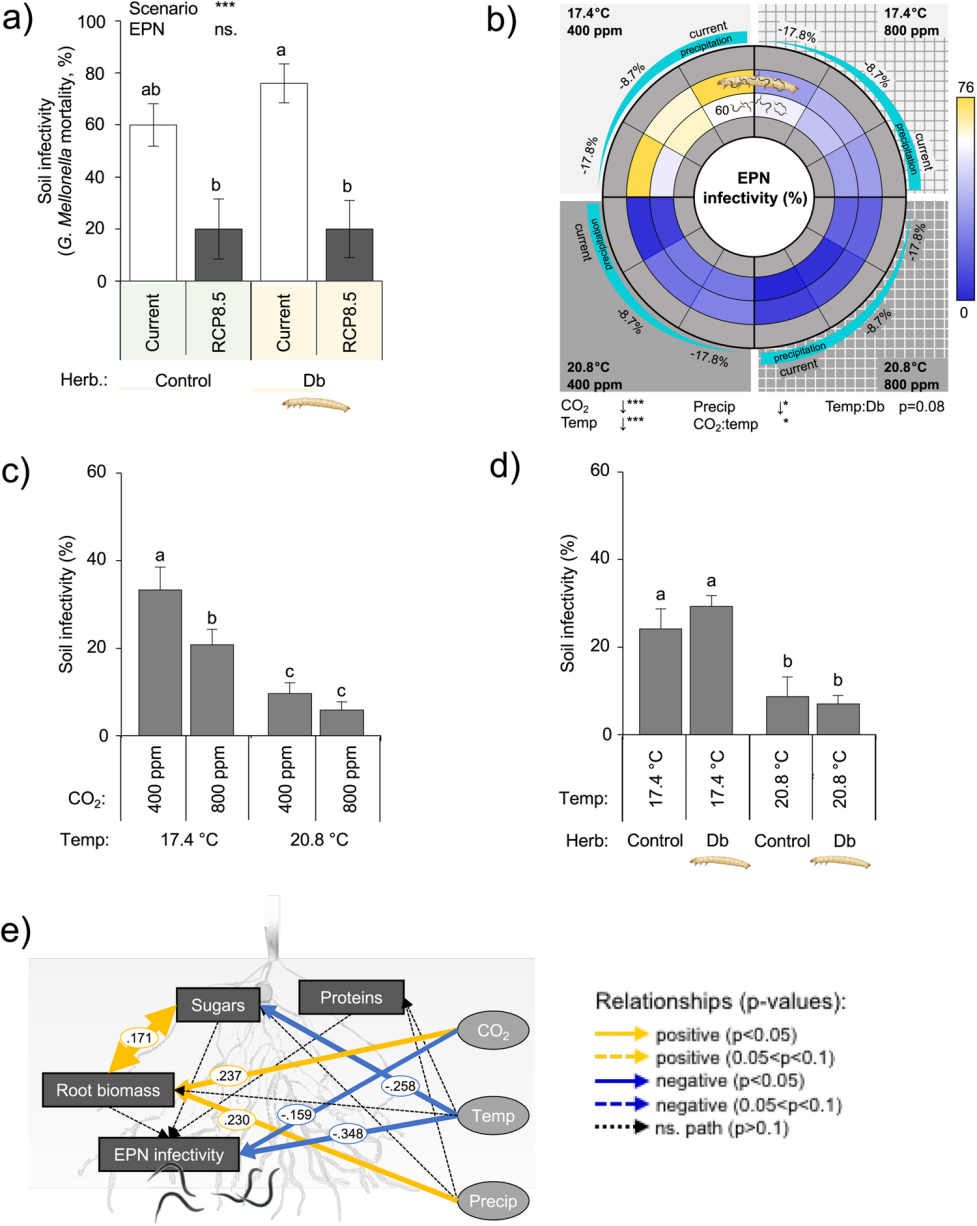
Entomopathogenic nematode (EPN) success in response to individual and combined abiotic factors. Plants subjected to herbivory were infested with six second-instar larvae of the herbivore *Diabrotica balteata*. A week later, half of the control and half of the herbivore-infested plants further received 2500 entomopathogenic nematodes, *Heterorhabditis bacteriophora*. EPN success was measured through baiting assays. Homogenized soil samples (80 g/pot) were collected and placed into solo cups containing five *Galleria mellonella* for 7 days. The infection status of *G. mellonella* was assessed 7 days later. **a** EPN infectivity under current and predicted climatic scenario RCP 8.5 (Collins et al. 2013). Current conditions: 400 ppm CO_2_, 17.4 °C, and 27.0% soil gravimetric water (“current”). RCP 8.5 conditions: 800 ppm CO_2_, 20.8 °C, and 22.2% soil gravimetric water. Average ± SEM are shown. **b** EPN infectivity in response to individual and combined abiotic factors. EPN infectivity was measured in the full factorial 24 combinations of CO_2_ (current: 400 ppm, RCP 8.5: 800 ppm), temperature (current: 17.4 °C, RCP 8.5: 20.8 °C), moisture (current: 27% gravimetric moisture, intermediate − 8.7% precipitation relative to current conditions (24.6% gravimetric moisture), RCP 8.5: − 17.8% precipitation relative to current conditions (22.2% gravimetric moisture)), and presence of the root herbivore *Diabrotica balteata* (Db), are presented with a spider net. The color code indicates the relative deviation of the averages from current climate conditions (white indicates current control conditions, blue and yellow respectively indicate reductions and increases of the responsive variable). The vertical separation indicates ambient versus elevated atmospheric CO_2_ conditions. The horizontal separation indicates high versus low soil temperatures. Each quarter follows a soil moisture gradient, corresponding to different precipitation predictions (current, − 8.7% and − 17.8%). Different circle layers correspond to the different biotic conditions, starting with the control treatments in the center (plant only), followed by plants + EPNs, plants + Db + EPNs, and plants + Db. Plants from only the two middle circles were infested by herbivores, thus the most inner and outer circles are shown in grey. Interactive effects of **c** temperature and CO_2_, and of **d** temperature and herbivore presence (Db). **e** Structural equation model of the relationships between major climate parameters (CO_2_, soil temperature and precipitation), plant responses and EPN infectivity. Single terms were included only. Arrow weights are proportional to standardised coefficients, which are indicated alongside the arrow. Blue arrows: negative relationships (continuous: p < 0.05, dashed: 0.05 < p < 0.10), yellow arrows: positive relationships (continuous: p < 0.05, dashed: 0.05 < p < 0.10), dashed black arrows: non-significant relationships, improving the models. *Temp* temperature, *Precip* precipitation, *Db* *Diabrotica balteata* (herbivore), *EPNs* entomopathogenic nematodes (herbivore enemies). Stars indicate a significant impact of the tested treatment. *p ≤ 0.05, **p ≤ 0.01, ***p ≤ 0.001). No interaction between treatments was noted. Different letters indicate significant differences (Tukey’s HSD: p ≤ 0.05). The arrows below the spider net indicate the direction of the response along the treatment gradient. All statistical results and model fit indices are shown in Table 1 and in Online Resource 2

Under the RCP 8.5 scenario, the efficacy of biological control agents was considerably impaired and dropped from 60 to 20% (Fig. 7a).

CO_2_, temperature, and moisture modulated EPN performance (Table 1). Elevated CO_2_ and temperature decreased EPN infectivity in baiting assays (Fig. 7b). On the contrary, decreased moisture enhanced EPN infectivity (Fig. 7b). CO_2_ and temperature had interactive effects, as elevated CO_2_ decreased infectivity under ambient, but not elevated temperatures (Fig. 7c). Finally, the temperature-dependent decrease in infectivity was stronger when the herbivore had been present in the root vicinity (Fig. 7d).

Structural equation modelling revealed strong negative effects of elevated CO_2_ and temperature levels on EPN infectivity, but no effects of root biomass or chemical composition (Fig. 7e). Thus, EPN infectivity seems to be influenced directly by abiotic factors rather than indirectly through plant biomass or chemistry.

## Discussion

This study illustrates the strong influence of climate change on the outcomes of tritrophic interactions and its detrimental impact on biological control. The changes in temperature, moisture, and CO_2_ directly modulated the metabolism/behavior of plants, herbivores, and their enemies, further altering the interactions between the different trophic levels. Interestingly, using a full factorial design involving different CO_2_, temperatures, and moisture levels, revealed that the climatic and herbivory effects were mostly additive. The possible mechanisms and (agro)ecological relevance of these findings are discussed below.

Single climatic variables had a strong impact on plant metabolism and growth. Yet, when combined, their effects mostly acted additively and counter-balanced each other. CO_2_ increased leaf biomass and decreased root benzoxazinoid contents. This is consistent with some previous work showing that CO_2_ elevation can lead to larger shoot biomass, even in C4 plants, while in most cases root growth was unaffected (Wand et al. 1999; Silva et al. 2020). CO_2_-driven changes of metabolite composition have previously been reported, but there is no clear trend as for the change in direction (Hiltpold et al. 2017). In maize, elevated CO_2_ was previously reported to decrease benzoxazinoid concentrations in shoots (Vaughan et al. 2014, 2016), and we observe a similar effect in roots. Higher temperatures were surprisingly associated with decreased leaf wilting, but also with decreased root sugar and benzoxazinoid contents, and increased biomass. Oppositely, lower soil moisture levels were associated with increased leaf wilting, sugar, and benzoxazinoid contents, and decreased biomass and protein contents. These effects were mostly consistent with the previous literature on elevated temperature and drought effects on plant growth (Lobell et al. 2014; Guyer et al. 2018; Lizaso et al. 2018) and physiology (Morison and Lawlor 1999; Jochum et al. 2007; Dyer et al. 2013), but these effects depend on the crop species and cultivation region (Hentley and Wade 2017). The direct impacts of CO_2_, temperature, and moisture mostly offset one another, resulting in neutral effects of the predicted climatic scenario RCP 8.5 on maize plant growth and physiology. For instance, the negative impact of the reduced soil moisture on the plant biomass could be counterbalanced at higher temperature and CO_2_, possibly through enhanced stomatal closure (Lopes et al. 2011; Manderscheid et al. 2014). Only the sharp negative impact of drought on leaf wilting was not completely compensated by other variables. Extended leaf wilting symptoms during flowering have been associated with a decreased yield in maize (Shin et al. 2015). Although our study focused on plants at the vegetative stage, it is likely that RCP 8.5 conditions would have resulted in a yield penalty. Merely one interaction between individual climatic factors was noted to affect plant growth. Specifically, temperature and moisture interacted in shaping the leaf biomass, as the latter decreased at lower soil moistures more rapidly at higher temperatures. This interactive effect was overridden by the strong impact of individual factors and their additive effects under the RCP 8.5 scenario. The plants used in this study were exposed to climatic conditions during the early developmental stage, and future studies are required to understand the impact of climate change on maize germination, reproduction, and senescence.

RCP 8.5 climatic conditions did not affect the plant response to herbivory. The impact of root herbivory on plants was overall similar between current and predicted scenarios. Root damage resulted in increased leaf wilting, reduced root biomass, and reconfigured the plant sugar metabolism. Benzoxazinoids were not induced by herbivory, a finding consistent with previous reports suggesting that root benzoxazinoids are constitutively present at high concentrations but not induced in roots (Robert et al. 2012). Yet, at this stage, potential differences in plant response to herbivory under climate change cannot be fully excluded. Recently, Paudel et al. showed for instance that temperature may influence the amount of the glucose oxidase salivary elicitor from caterpillars, resulting in differences in plant defense and resistance to herbivory (Paudel et al. 2020). More investigations of how climate change will modulate the induced plant response to herbivory are crucially needed.

Predicted future climatic scenarios benefited the root herbivore. CO_2_, temperature, their interactions with soil moisture, and the presence of EPNs were the main determinant of the root herbivore performance and survival. Under the RCP 8.5 scenario, the herbivore performed better, although its survival tended to decrease in absence of natural enemies in the soil. The full factorial analysis revealed a positive impact of elevated CO_2_ and temperature on the insect mass. The impact of CO_2_ on herbivore performance seems to be both direct and indirect, through a reduction of sugar and benzoxazinoid concentrations in plants. Ectotherm species are sensitive to temperature changes and warming is likely to promote their feeding activity and performance (Gregory et al. 2009; DeLucia et al. 2012). Yet, as root damage was similar between the different conditions, it is likely that the mass differences are due to either direct physiological changes in the insect food processing mechanisms or to indirect changes through un-measured metabolic differences in the plants. Change of tissue quality, including nutrient availability and secondary metabolites, interferes with herbivore performance (DeLucia et al. 2012; Erb and Lu 2013; Lin et al. 2021) and feeding activity (Bale et al. 2002; Levesque et al. 2002; Golizadeh et al. 2007). Despite the direct link between benzoxazinoid concentrations and generalist herbivore survival (Wouters et al. 2016), the observed, albeit non-significant, decrease in benzoxazinoid concentrations under the RCP 8.5 scenario did not result in enhanced survival of the herbivore. Interestingly, the SEM even reported a positive correlation between benzoxazinoid contents and herbivore survival in absence of entomopathogenic nematodes in the rhizosphere. Exuded DIMBOA is known to chelate with iron in the soil, a complex that can be highjacked by the root herbivore *D. virgifera* for its own nutrition (Hu et al. 2018a). Whether *D. balteata* would be using a similar nutritive strategy that would explain a better survival remains to be tested. Benzoxazinoids can be detoxified and sequestered by *D. virgifera*, conferring the latter increased resistance to EPN infection. Yet, *D. balteata* larvae are not able to use benzoxazinoids for their protection from EPNs, which may explain the fact that benzoxazinoid contents are not correlated with survival in presence of EPNs in soil (Robert et al. 2017). Although RCP 8.5 did not significantly reduce herbivore survival, warmer temperatures directly impaired *D. balteata* survival. Heat may impair herbivore survival through a series of alterations in molecular, biochemical, and physiological processes, including protein denaturation, cellular homeostasis imbalance, neurophysiological functioning limitation, or shifts in endosymbiont populations (Ma et al. 2021). Heat may also threaten herbivore survival indirectly, through water loss and desiccation (Chown et al. 2011)*.* As the impact of temperature on the herbivore survival was accentuated under drought, it is likely that the insects suffered from desiccation under the RCP 8.5 scenario. The thresholds of temperature and moisture levels that significantly impair the herbivore survival in the field would be interesting to characterize in order to better predict herbivore population dynamics. Yet, under these thresholds, the pest insect will benefit from climate change. The consequences of the increased insect performance on the insect fitness and phenology remain to be assessed in the field, to evaluate its contribution to potential outbreaks.

Climate change drastically impeded the efficacy of entomopathogenic nematodes as biological control agents. Although EPNs significantly reduced the survival of the root herbivore in all tested climatic combinations, their efficacy as biological control agents dropped from 60 to 20% under the RCP 8.5 scenario. Elevated temperatures and CO_2_ directly impeded the EPN infectivity potential. Elevated CO_2_ may directly interfere with EPN host location ability, as most EPNs rely on CO_2_ gradients to locate a host (Zhang et al. 2021; Hallem et al. 2011; Dillman et al. 2012). Hiltpold et al. (2020) also observed a negative impact of elevated CO_2_ on *H. bacteriophora* but inferred this effect to differences in root morphological complexity, which would, in turn, impede EPN efficiency in searching for a host (Demarta et al. 2014). Although the impact of CO_2_ on EPNs seems to be consistent, the underlying mechanisms remain to be tested. This strong negative impact of elevated CO_2_ on EPN infectivity at current temperatures disappeared under elevated temperatures. Indeed, the negative effect of temperature suggests that 20.4 soil temperature was above the optimal temperature for *H. bacteriophora* (Pervez et al. 2016). Warm environmental conditions lead to higher body energy consumption and water loss from the soft-bodied parasites, which may explain the observed reduction in infection potential (Glazer 2002). Yet, lower moisture levels improved EPN infectivity. While this result may be surprising given the fact that EPNs require a water layer in the soil for movement (Salame and Glazer 2015), it has been previously reported in the literature (van Doan et al. 2021). However, structural equation modelling suggests that the impact of moisture on EPN infectivity was not direct, but rather plant- and/or herbivore-mediated. The role of plant exudates in the survival of EPNs has been suggested (Zhang et al. 2021). It is interesting to note that the presence of EPNs itself triggered a plant response (larger root system and lower protein contents) independently of the climatic conditions. Furthermore, the presence of EPNs modulated the interactions between the plants and their herbivores. Altogether, these observations point towards some overlooked impact of EPNs on plant physiology and associated feedbacks onto plant–herbivore interactions (Zhang et al. 2021; Jagdale et al. 2009; An et al. 2016; Helms et al. 2019). The negative direct and indirect effects of climate change on EPN success underlines the urge to adapt biological control strategies, for instance through the use of EPNs from warmer, dryer, regions, or through the selection of EPNs tolerant to predicted climatic conditions, to prevent pest outbreaks in the future (Baïmey et al. 2017; Glazer 2002; Stock et al. 2008).

In conclusion, this study highlights that climate-associated abiotic factors strongly modulate multi-trophic interactions. While the predicted RCP 8.5 scenario will likely impede maize yield and biological control agent efficiency, it will enhance the herbivore performance, possibly favoring soil-dwelling pest outbreaks in Central Europe. Abiotic and biotic parameters acted additively and interactively on the system, underlining the importance of integrating several factors in future studies. Furthermore, the effects of climate change were both direct and indirect, emphasizing the need to combine several trophic levels together in building predictive models about the impact of climate change on agro-ecosystems.

## Supplementary Information

Below is the link to the electronic supplementary material.

Supplementary file1 (PDF 191 kb)

Supplementary file2 (DOCX 25 kb)

Supplementary file3 (PDF 228 kb)

Supplementary file4 (PDF 589 kb)

Online Resource 5. Raw data. (XLSX 58 kb)

Supplementary file6 (DOCX 13 kb)

## Data Availability

Raw data are available as an online (Online Resource 5).

## Code Availability

R codes are available as an online (Online Resource 6).
