# Supplementary material for: Climate Change Modulates Multitrophic Interactions Between Maize, A Root Herbivore, and Its Enemies
Source: J Chem Ecol. 2021 Aug 20;47(10-11):889–906. doi: 10.1007/s10886-021-01303-9 (PMC8613123; doi:10.1007/s10886-021-01303-9)
Supplement: Supplementary file 2 — Supplementary file2 (DOCX 25 kb) [file 10886_2021_1303_MOESM2_ESM.docx]

**ONLINE RESOURCE 2:** Detailed information on unstandardized path coefficients, standard errors (SE), p-values and standardized path coefficients presented in the Figures. Abbreviations: SEM: structural equation modelling, T: soil temperature, M: soil moisture, CO_2_: atmospheric CO_2_, EPN: entomopathogenic nematodes, Unstd: unstandardized, Std: standardized. Values in bold indicate significant (p < 0.05) paths.

Detailed information on the SEM from Figure 6a

|  |  | Coefficients (Unstd.) | SE | Coefficients (Std.) | p-value |
| --- | --- | --- | --- | --- | --- |
| Regressions: |  |  |  |  |  |
| BXDs | CO2 | -0.077 | 0.232 | -0.136 | 0.557 |
|  | T | -0.245 | 0.233 | -0.437 | *0.060* |
|  | M | -0.193 | 0.141 | -0.208 | 0.138 |
| Sugars | CO2 | -0.140 | 0.094 | -0.101 | 0.282 |
|  | T | -0.188 | 0.095 | -0.136 | 0.149 |
|  | M | -0.223 | 0.057 | -0.098 | *0.087* |
| Db survival | T | -0.355 | 0.051 | -0.154 | **0.003** |
|  | M | 0.151 | 0.031 | 0.040 | 0.198 |
|  | BXDs | 0.317 | 0.030 | 0.077 | **0.010** |
|  | Sugars | 0.102 | 0.073 | 0.061 | 0.401 |
| Covariance: | BXDs ~ Sugars | 0.245 | 0.041 | 0.071 | *0.083* |

Detailed information on the SEM from Figure 6b

|  |  | Coefficients (Unstd.) | SE | Coefficients (Std.) | p-value |
| --- | --- | --- | --- | --- | --- |
| Regressions: |  |  |  |  |  |
| BXDs | CO2 | -0.244 | 0.265 | -0.528 | **0.046** |
|  | T | -0.253 | 0.265 | -0.545 | **0.039** |
|  | M | -0.205 | 0.159 | -0.266 | *0.095* |
| Sugars | CO2 | -0.279 | 0.065 | -0.168 | **0.010** |
|  | T | -0.481 | 0.065 | -0.290 | **0.000** |
|  | M | -0.179 | 0.039 | -0.065 | *0.098* |
| Db survival | T | -0.252 | 0.067 | -0.116 | *0.084* |
|  | M | -0.169 | 0.036 | -0.047 | 0.194 |
|  | BXDs | 0.229 | 0.030 | 0.049 | 0.108 |
|  | Sugars | -0.348 | 0.120 | -0.265 | **0.028** |
| Covariance: | BXDs ~ Sugars | 0.289 | 0.033 | 0.068 | **0.040** |

Detailed information on the SEM from Figure 6c

|  |  | Coefficients (Unstd.) | SE | Coefficients (Std.) | p-value |
| --- | --- | --- | --- | --- | --- |
| Regressions: |  |  |  |  |  |
| Db survival | CO2 | -0.017 | 0.037 | -0.008 | 0.827 |
|  | T | -0.275 | 0.037 | -0.135 | **0.000** |
|  | M | -0.035 | 0.023 | -0.011 | 0.647 |
|  | EPN | -0.479 | 0.037 | -0.236 | **0.000** |
| Root damage | CO2 | 0.030 | 0.329 | 0.110 | 0.739 |
|  | T | 0.185 | 0.342 | 0.684 | **0.046** |
|  | M | 0.144 | 0.202 | 0.328 | 0.104 |
|  | Db survival | 0.061 | 0.696 | 0.458 | 0.510 |
| Root biomass | CO2 | 0.038 | 0.103 | 0.050 | 0.626 |
|  | T | 0.267 | 0.108 | 0.355 | **0.001** |
|  | M | 0.261 | 0.064 | 0.213 | **0.001** |
|  | Db survival | 0.078 | 0.217 | 0.211 | 0.332 |
|  | Root damage | -0.484 | 0.028 | -0.174 | **0.000** |

Detailed information on the SEM from Figure 7e

|  |  | Coefficients (Unstd.) | SE | Coefficients (Std.) | p-value |
| --- | --- | --- | --- | --- | --- |
| Regressions: |  |  |  |  |  |
| Root biomass | CO2 | 0.153 | 0.117 | 0.237 | **0.043** |
|  | T | 0.143 | 0.144 | 0.221 | 0.125 |
|  | M | 0.243 | 0.088 | 0.230 | **0.009** |
| Sugars | T | -0.301 | 0.080 | -0.258 | **0.001** |
|  | M | -0.112 | 0.049 | -0.059 | 0.230 |
| Proteins | T | 0.112 | 0.143 | 0.165 | 0.250 |
|  | M | -0.063 | 0.234 | -0.151 | 0.250 |
| EPN infectivity | CO2 | -0.260 | 0.044 | -0.159 | **0.000** |
|  | T | -0.570 | 0.048 | -0.348 | **0.000** |
|  | Proteins | -0.018 | 0.018 | -0.005 | 0.796 |
|  | Sugars | 0.14 | 0.062 | 0.1 | 0.105 |
|  | Root biomass | -0.119 | 0.034 | -0.047 | 0.162 |
| Covariance: | Root biomass ~ Sugars | 0.579 | 0.034 | 0.171 | **0.000** |
