# Supplementary material for: Climate Change Modulates Multitrophic Interactions Between Maize, A Root Herbivore, and Its Enemies
Source: J Chem Ecol. 2021 Aug 20;47(10-11):889–906. doi: 10.1007/s10886-021-01303-9 (PMC8613123; doi:10.1007/s10886-021-01303-9)

Climate change remodulates multitrophic interactions between maize, a root herbivore, and its enemies

Anouk Guyer[1,2], Cong Van Doan[1,2], Corina Maurer[1], Ricardo A.R. Machado[1], Pierre Matéo[1], Katja Steinauer[1], Lucie Kesner[1], Günter Hoch[3], Ansgar Kahmen[3], Matthias Erb[1,2], Christelle AM Robert[1,2*]

[1] *Institute of Plant Sciences, University of Bern, Altenbergrain 21, 3013 Bern, Switzerland.*
[2] *Oeschger Centre for Climate Change Research (OCCR), University of Bern, Falkenplatz 16, 3012 Bern, Switzerland.*
[3] *Department of Environmental Sciences - Botany, University of Basel, Schönbeinstrasse 6, 4056 Basel, Switzerland.*

[*] Corresponding author: christelle.robert@ips.unibe.ch

ONLINE RESOURCE 4

Additional structural equation models about factors modulating the herbivore survival

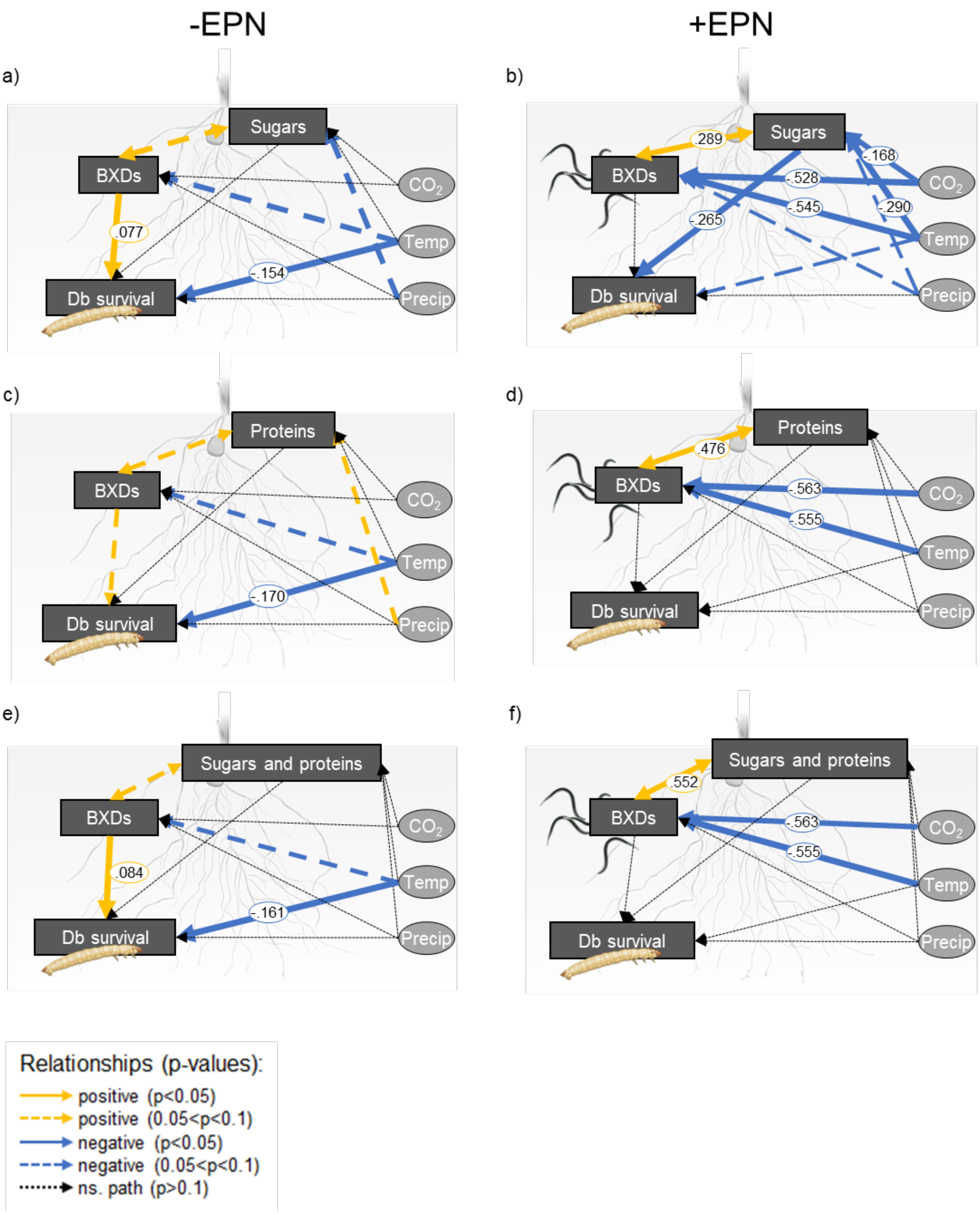

Supplement: Supplementary file 4 — Supplementary file4 (PDF 589 kb) [file 10886_2021_1303_MOESM4_ESM.pdf]
