# Supplementary material for: Climate Change Modulates Multitrophic Interactions Between Maize, A Root Herbivore, and Its Enemies
Source: J Chem Ecol. 2021 Aug 20;47(10-11):889–906. doi: 10.1007/s10886-021-01303-9 (PMC8613123; doi:10.1007/s10886-021-01303-9)
Supplement: Supplementary file 6 — Supplementary file6 (DOCX 13 kb) [file 10886_2021_1303_MOESM6_ESM.docx]

**R codes**

**Linear models:**

*Example:*

library(RVAideMemoire)

library(car)

library(multcomp)

mod.wilting_sc <- lm( wilting_sc ~ CO2+Db+EPN, data=data.pool)

plotresid(mod.wilting_sc)

Anova(mod.wilting_sc)

wilting_sc.lsm <- lm( wilting_sc ~ group, data=data.pool)

lsm.wilting_sc = lsmeans(wilting_sc.lsm,~ group)

cld(lsm.wilting_sc, alpha=0.05, Letters=letters, adjust="tukey")

**Structual equations models:**

*Example:*

library(lavaan)

library(PerformanceAnalytics)

library(semTools)

library(semPlot)

sem.EPN.4 <- "

FW.root_g ~ CO2+temp+moist

sugars.mg.g ~ moist+temp

Protein.mg.g ~ moist+temp

gall.inf_pct ~ CO2+temp+Protein.mg.g+sugars.mg.g+FW.root_g

FW.root_g ~~ sugars.mg.g

"

sem.fit.EPN.4 <- cfa(sem.EPN.4, data=SEM_withEPN) #, test="bootstrap")

summary(sem.fit.EPN.4, standardized=TRUE, rsquare=TRUE)

fitmeasures(sem.fit.EPN.4, fit.measures = c("pvalue", "df", "chisq", "RMSEA", "SRMR", "CFI", "GFI"))
